# 

**Published:** 1897-02

**Authors:** 


					﻿CONTENTS.
ORIGINAL ARTICLES—
Hypertrophic E'ongation of the Cervix Uteri, With Complete
Eversion of Vagina, From Fibroid Tumor of the Cervix, Res-
toration by Operation. By George Henry Noble, M. D., At-
lanta, Ga............................................... 53
Primary Lateral Sclerosis. By S. G. Courtney Pinckney, M. D.,
Atlanta, Ga...........................................   58
Uterine Drainage as a Factor in the Prevention and Relief of
Pelvic Inflammation. By R. R. Kime, M. D., Atlanta, Ga.. 59
Retinal Hemorrhage Caused by Lithemia. By A. Bethune Pat-
terson, M. D., Atlanta, Ga.............................. 65
A Short Lecture Delivered to the Students of the Atlanta Medical
College, on Influenza. By W. S. Kendrick, M. D., Atlanta, Ga.. 68
SELECTIONS AND ABSTRACTS—
The Janet Abortive Treatment of Gonorrhea............... 73
Surgery of the Appendix ...............................  74
A Simple Method of Treating Opium Poisoning............. 76
The Anatomy of Rape............................  :...... 76
Surgical Hints.......................................... 77
Early Signs of Locomotor Ataxia......................... 78
The Modern Treatment of Typhoid.......................    79
Hydrozone in Gastric and Intestinal Disorders..........   80
The Demand and Supply of Eunuchs in China................ 82
The Feeding of the Sick................................   83
Catching Cold...........;..............................   85
Cosmetics of Castration .. .....•.......................  85
Dacryocystitis and Its Treatment........................  86
Notes on the Treatment of Foecal Fistula;................ 87
The Indications for Ventral Suspension of the Uterus .... 88
, The Suture-Clamp Operation for Hemorrhoids.. ........... 89
Epithilial Sowing—A New Method of Skin-Grafting.......... 90
Proper Management of Appendicitis........................ 91
Massage in the Treatment of Muscular Rheumatism.......... 92
Treatment of Detachment of the Retina.................... 93
Peculiar Virtues of Cod Liver Oil....................     93
Cocaine Poisoning—Magnan’s Symptoms.......................94
EDITORIAL..................................................  95
BOOK REVIEWS................................................ 97
				

